# Exploring Age and Gender Identification Through Mandibular Parameters Using Orthopantomography: An Observational Study

**DOI:** 10.7759/cureus.55788

**Published:** 2024-03-08

**Authors:** Abirami Arthanari, Akshai Senthilkumar, Karthikeyan Ramalingam, Lavanya Prathap, Vignesh Ravindran

**Affiliations:** 1 Department of Forensic Odontology, Saveetha Dental College and Hospitals, Saveetha Institute of Medical and Technical Sciences, Saveetha University, Chennai, IND; 2 Department of Oral Pathology and Microbiology, Saveetha Dental College and Hospitals, Saveetha Institute of Medical and Technical Sciences, Saveetha University, Chennai, IND; 3 Department of Anatomy, Saveetha Medical College and Hospitals, Saveetha Institute of Medical and Technical Sciences, Saveetha University, Chennai, IND; 4 Department of Pediatric Dentistry, Saveetha Dental College and Hospitals, Saveetha Institute of Medical and Technical Sciences, Saveetha University, Chennai, IND

**Keywords:** ramus breadth, dimorphic bone, gonial angle, coronoid height, condylar height, projective ramus, condylar ramus, mandibular metrics, sex determination, age estimation

## Abstract

Aim

This study aims to examine five mandibular parameters: coronoid ramus height, condylar ramus height, projective ramus height, minimum ramus breadth, and gonial angle, using orthopantomography (OPG).

Introduction

The mandible, a crucial part of the human skull, demonstrates sexual dimorphism, which makes it an important tool for determining sex in forensic and anthropological investigations. Its form and structure are relatively resistant to significant changes after death. Among all skeletal components, the mandible stands out as a primary indicator of sexual differences. In forensic investigations, establishing the age and sex of an individual is considered a crucial initial step. This process can be particularly challenging in scenarios involving mass casualties, natural calamities, or extensively fragmented remains. Due to its responsiveness to growth patterns, the mandible is adept at accurately determining both age and sex.

Materials and methods

This study employed a sample size of 500 individuals, split equally between males and females, with 250 participants each. The age bracket chosen for this cohort ranged from 20 to 30 years, considering that bone growth characteristics within this range can assist in sex determination. The height of the mandibular ramus was assessed using Planmeca software, and subsequent data analysis was conducted using SPSS.

Results

When estimating age, the condylar ramus height exhibited the smallest standard error (0.010), whereas the maximum standard error for the gonial angle was 0.028. Positive t values were observed for the gonial angle (1.182), minimum ramus breadth (0.114), and coronoid ramus height (0.733). In terms of determining sex, the gonial angle, coronoid ramus height, and projective ramus height demonstrated positive coefficient functions, specifically 0.676, 0.090, and 0.286, respectively. Conversely, both the minimum ramus breadth and the condylar ramus height displayed negative values of -0.385 and -0.126, respectively.

Conclusion

Among the parameters evaluated, condylar ramus height emerges as the most suitable choice for estimating age, while gonial angle, coronoid ramus height, and projective ramus height are preferable for determining sex.

## Introduction

Determining the age and gender of a person is one of the most challenging aspects of forensic dentistry. In the modern world, human identification during post-mortem profiling is easily accomplished with a database [1]. To identify dental and skeletal remains at the scene of crime, explosions, warfare, aircraft crashes, catastrophic disasters, and missing individuals, dentists are increasingly playing an important role in identifying the individual. The skull, pelvic bone, and cranium are the essential skeletal tools that can be used for identification [2]. The mandible is the most unusual and resilient bone. It can be readily removed from the accident scene with little damage and still retains its natural shape. A person's age and gender can also be determined with the use of mandibular parameters [3]. The gonial angle, mental form, and mandibular shape are a few examples of anthropometry tools used in sex determination techniques [4].

Normally, the age and sex of an unknown individual can be identified based on the metric features of the mandible as well. Male and female mandibular parameters are distinguished according to their shape, size, angle, and flares. Loth et al. have observed major changes in the angulation of a mandible between males and females and proved that mandibular parameters are essential tools to identify an individual’s age and sex [5].

A panoramic radiograph is very useful in displaying the entire mandibular and maxillary structures on a single film; hence, it has an additional advantage over other extra-oral radiographs [6]. This study reports the correlation, analysis, and evaluation of the mandibular parameters using an orthopantomogram. The objective of this study is to examine five mandibular parameters: coronoid ramus height, condylar ramus height, projective ramus height, minimum ramus breadth, and gonial angle using orthopantomography (OPG).

While assessing various populations and races, the variations in the coronoid process of the mandible can function as anthropological markers. The enlarged coronoid process in male mandibular morphology may be influenced by the extensive muscle mass of the temporalis [3]. The coronoid ramus height represents the projected distance from the lower wall of the bone to the coronion point. The measurement of the condylar ramus height involves determining the distance from the highest point of the mandibular condyle to either the tubercle or the most prominent part of the lower border of the ramus [7]. The distance measured from the lower edge of the bone to the highest point of the mandibular condyle is the ramus's projected height [8]. Minimum length of horizontal space between the ramus's anterior and posterior points or the minimum width of the mandibular ramus, as measured perpendicularly to its height [9]. The mandibular base's shape is greatly influenced by the structure and function of the masticatory muscles, particularly the gonial angle. Regarding age, contraction, and muscle density also decrease [6]. This study aims to examine five mandibular parameters: coronoid ramus height, condylar ramus height, projective ramus height, minimum ramus breadth, and gonial angle, using orthopantomography (OPG) to determine the gender of an individual.

## Materials and methods

**Figure 1:**
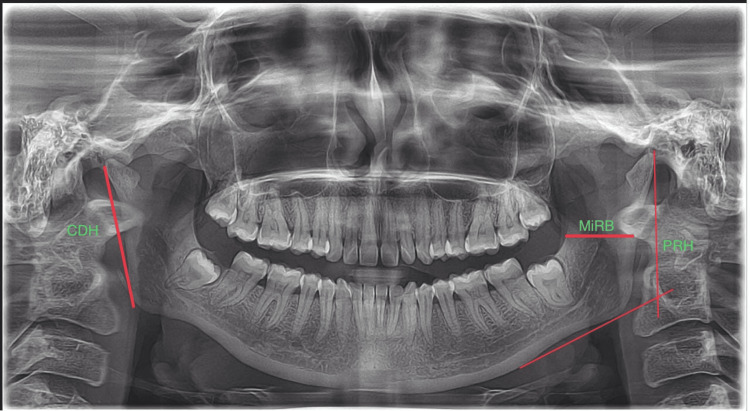
Mandibular parameters selected for this study CDH: condylar ramus height; MiRB: minimum ramus breadth; PRH: projective ramus height

The study was conducted on randomly selected 500 samples (250 males and 250 females), aged between 20 and 30 years, using standard digital OPG. Cases with known sex and the availability of orthopantomogram radiographs for each sample with head alignment that was contrasting and clearly visible were included in the study. Radiographs that indicated any pathological lesions and other artifacts were excluded from the study. The collected radiographs were archived in the Department of Oral Medicine and Radiology, Saveetha Dental College and Hospitals, Chennai. The Institutional Human Ethics Committee of Saveetha Dental College (IHEC/SDC/FACULTY/22/FO/059) approved this study. The sample size was calculated to ensure a statistical power of 95% and a significance level (alpha error probability) of 0.05, employing G-Power software (version 3.1.9.4, Düsseldorf, Germany). The calculated sample size was 482, and we have included a total of 500 samples. The software used to analyze the samples is Planmeca (Planmeca Romexis®, Version 6.0, United States), and was used to measure the height of the mandibular ramus. The obtained data was analyzed using IBM SPSS Statistics for Windows, Version 16 (released 2007; IBM Corp., Armonk, New York, United States). The parameters like condylar ramus height, minimum ramus breadth, and projective ramus height were measured in the present study, as represented in Figure 1.

**Figure 2:**
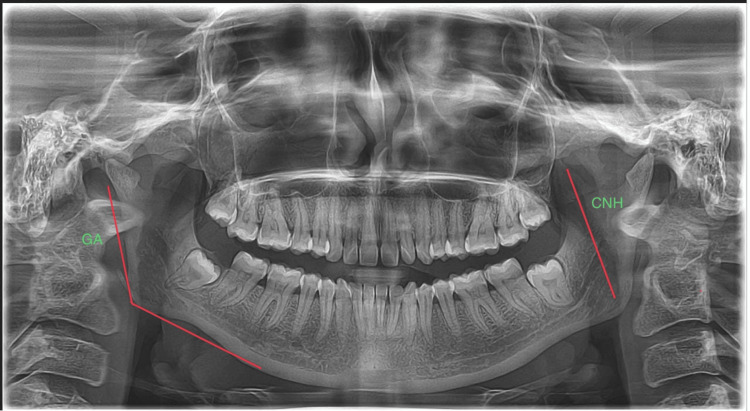
Mandibular parameters selected for this study CNH: coronoid ramus height; GA: gonial angle

The parameters like coronoid ramus height and gonial angle were measured in the present study as represented in Figure 2. 

## Results

Following a review of the means for each gender, the lowest and maximum mean values were shown. By using an independent "t" test on the data and determining the "p" value, the significance of the parameters in both males and females was determined. Furthermore, the standard deviation (SD) for each gender was assessed with a linear coefficient of age, representing the standard error of the estimate as given in Table 1.

**Table 1:** Linear coefficient of age with standard error of the estimate

Model	R	R Square	Adjusted R Square	Std. Error of the Estimate
1	0.087^a^	0.008	-0.011	3.227

The coefficient of the dependent variables between regression and residual analysis was done as represented in Table 2.

**Table 2:** Coefficient of dependent variables between regression and residuals A. dependent variable: age; B. predictors: (constant), gonial angle (GA) (degree), minimum ramus breadth (MiRB) (mm), condylar ramus height (CDH) (mm), coronoid ramus height (CNH) (mm), projective ramus height (PRH) (mm)

Model		Sum of Squares	df	Mean Square	F	Sig.
1	Regression	39.175	9	4.353	0.418	0.926^b^
	Residual	5102.913	490	10.414	-	-
	Total	5142.088	499	-	-	-

Based on the discriminant analysis as represented in Table 3, the following formulae have been derived based on the measured parameters.

**Table 3:** Discriminant analysis of all parameters

Model	Unstandardized coefficient		Standardized coefficient		
	B	Std. Error	Beta	t	Sig.
Projective ramus height (PRH) (mm)	-0.019	0.020	-0.046	-0.962	0.337
Minimum ramus breadth (MiRB) (mm)	0.003	0.026	0.005	0.114	0.909
Gonial angle (GA) (mm)	0.033	0.028	0.054	1.182	0.238
Condylar ramus height (CDH) (mm)	-0.003	0.010	-0.013	-0.291	0.772
Coronoid ramus height (CNH) (mm)	0.015	0.020	0.034	0.733	0.464

**Figure 3:**
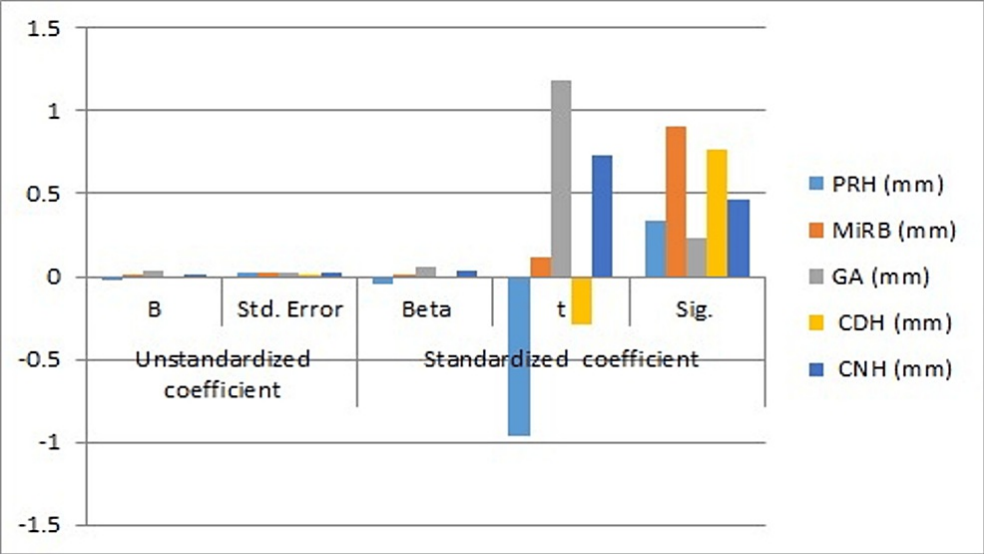
Graph showing standardized and unstandardized coefficient values, t, and significance values PRH: projective ramus height; MiRB: minimum ramus breadth; GA: gonial angle; CDH: condylar ramus height; CNH: coronoid ramus height

The formula derived for age determination using projective ramus height (PRH) is age = 22.709-0.019 PRH. The formula derived for age determination using minimum ramus breadth (MiRB) is age = 22.709+0.003 MiRB. The formula derived for age determination using gonial angle (GA) is age = 22.709+0.033 GA. The formula derived for age determination using coronoid ramus height (CNH) is age = 22.709+0.015 CNH. The formula derived for age determination using condylar ramus height (CDH) is age = 22.709-0.003 CDH. The least standard error of 0.010 was found for condylar ramus height. It was discovered that the gonial angle had the largest standard error, at 0.028. For both the condylar and projective ramus heights, a negative t value was noted. Positive t values were found for the gonial angle, minimum ramus breadth, and coronoid ramus height, respectively: 1.182, 0.114, and 0.733 (Figure 3).

The projective ramus height, gonial angle, and coronoid ramus height displayed positive coefficient functions of 0.676, 0.090, and 0.286, respectively, out of all the parameters taken into consideration (Table 4). Condylar ramus height and minimum ramus breadth both displayed negative values of -0.126 and -0.385, respectively. Among the parameters assessed, condylar ramus height is identified as the most appropriate for age estimation, while gonial angle, coronoid ramus height, and projective ramus height are favored for determining sex (Table 4).

**Table 4:** Standardized canonical discriminant of the measured parameters

Function Coefficients	Function
Projective ramus height (PRH) (mm)	0.676
Minimum ramus breadth (MiRB) (mm)	-0.385
Gonial angle (GA) (mm)	0.090
Condylar ramus height (CDH) (mm)	-0.126
Coronoid ramus height (CNH) (mm)	0.286

The following formula was thus derived from the above data:

Gender = -0.126*CDH+0,286*CNH+0.676*PRH- 0.385*MiRB+0.090*GA.

## Discussion

When estimating age, condylar ramus height showed the least standard error (0.010). In terms of the gonial angle, the maximum standard error was found to be 0.028. As for the gonial angle, minimum ramus breadth, and coronoid ramus height, positive t values of 1.182, 0.114, and 0.733 were noted, respectively. In determining sex, the gonial angle, coronoid ramus height, and projective ramus height all had positive coefficient functions, i.e., 0.676, 0.090, and 0.286, respectively. Both the minimum ramus breadth and the condylar ramus height showed negative values of -0.385 and -0.126, respectively.

Determining a victim's gender using human skeletal remains is regarded as the first stage in disaster victim identification [10]. It might not be possible to establish 100% accurate human identification in cases of mass disasters where the only remnants discovered are skeletal remains or fragments [11]. Establishing age and sex through anthropometric measurements of mandibular features is a significant area of research in forensic anthropology [12]. The biological profile of a person can be ascertained from their skeletal remains using this method, which is exact and accurate [13]. During an individual's life, the mandible undergoes distinct morphological changes that are utilized to determine their age, making it one of the primary skeletal components used for this purpose. The fact that its natural sexual dimorphism provides a foundation for sex determination accentuates its significance as a biometric tool. Sexual dimorphism is indicated by features of the mandible that highlight the distinct appearances between males and females [13,14]. It is customary to use factors like the presence of specific morphological traits and the size of the mandible to estimate a person's sex. The mandibular angle and chin both exhibit sexual dimorphism. Male chins generally appear more prominent and square-shaped, whereas female chins are often rounder and less apparent. These anatomical variations increase the accuracy of sex determination using mandibular characteristics [15]. The precision of age and sex determination using mandibular characteristics depends in part on the availability of large databases and standards for diverse populations, as variations may arise due to genetic, environmental, and nutritional factors [16].

This study looks at the significance, methods, and advancements in the application of mandibular parameters for anthropometric age and sex assessment. Recognizing that anthropometry is a non-destructive method that preserves skeletal integrity, it is particularly helpful in forensic settings where skeletal remains are frequently valuable and delicate [3]. This research examined mandibular parameters, encompassing both linear and angular measurements. Orthopantomogram (OPG) images were utilized for evaluation. The study compared bilateral measurements of the gonial angle, minimum ramus width, coronoid ramus height, condylar ramus height, and projective ramus height. The distance from the lowest point of the mandible to the highest point of the mandibular condyle defines the projective height of the ramus [17]. Variations in this dimension may manifest sexual dimorphism, which is useful in determining an individual's sex. The minimum width of the ramus indicates the narrowest horizontal span between its anterior and posterior points. This parameter is crucial to dental anthropology, forensic investigations, and anatomical studies.

The term "gonial angle" describes the angle created by two tangents drawn to the distal border of the ascending ramus and the lower border of the mandible [17,18]. Bone resorption and other aging-related processes lead to changes in the gonial angle. The distance spanning from the condyle of the ramus to its highest point on the lower border is denoted as the condylar ramus height [19]. The vertical distance extending from the coronoid process to the most conspicuous point along the lower edge of the ramus is designated as the coronoid-ramus height. According to research by Leversha et al., men exhibit greater ramus height and bigonial width compared to women [12,19]. According to Ojha B et al., females had greater gonial angles and maximum ramus breadths on both sides than males [20]. Compared to females, men frequently display larger angles. This differentiation is helpful in forensic investigations when determining sex [20,21].

OPG can provide measurements for the geometric and perpendicular dimensions of craniofacial features, although Akcam et al. argue that the precision of this data is inferior to that obtained from a lateral cephalogram [22]. Research carried out by Shahabi et al. demonstrated that panoramic radiography provided comparable accuracy to a lateral cephalogram in assessing the gonial angle [23].

Using mandibular characteristics alone has its limitations, just like any other age prediction method. Population, sex differences, interobserver variability, and postmortem modifications are a few of these restrictions. To improve the accuracy and reliability of age estimation in forensic contexts, forensic anthropologists and researchers frequently advise using multiple skeletal indicators and incorporating additional information, such as dental development, epiphyseal fusion, and other skeletal features. This is because of these limitations.

## Conclusions

In forensic and anthropological contexts, the mandible is an invaluable skeletal part for sex determination since it provides a range of morphological traits suggestive of sex dimorphism. Comprehending and employing these characteristics via meticulous examination techniques is crucial for precise sex determination from skeletal remains, supporting medico-legal probes, and anthropological studies.

An orthopantomogram (OPG) was utilized to analyze variables including coronoid ramus height, condylar ramus height, projective ramus height, minimum ramus breadth, and gonial angle across 500 individuals. Within the limitations of the present study, it can be concluded that the morphometric analysis of the mandibular ramus using OPG is a useful tool for sex determination and age estimation. Among the parameters evaluated, condylar ramus height emerges as the most suitable choice for estimating age, while gonial angle, coronoid ramus height, and projective ramus height are preferable for determining sex.
